# Understanding the Differences in the Growth and Toxin Production of Anatoxin-Producing *Cuspidothrix issatschenkoi* Cultured with Inorganic and Organic N Sources from a New Perspective: Carbon/Nitrogen Metabolic Balance

**DOI:** 10.3390/toxins12110724

**Published:** 2020-11-19

**Authors:** Siyi Tao, Suqin Wang, Lirong Song, Nanqin Gan

**Affiliations:** 1 State Key Laboratory of Freshwater Ecology and Biotechnology, Institute of Hydrobiology, Chinese Academy of Sciences, Wuhan 430072, China; tsy_0715@126.com (S.T.); lrsong@ihb.ac.cn (L.S.); 2 University of Chinese Academy of Sciences, Beijing 100049, China; 3 Hunan Key Laboratory for Health Aquaculture and Product Processing in Dongting Lake Area, Hunan University of Arts and Science, Changde 415000, China; suqinwanghn@outlook.com

**Keywords:** anatoxin-a, *Cuspidothrix issatschenkoi*, nitrogen sources, urea, L-alanine, C/N ratio

## Abstract

Cyanotoxins are the underlying cause of the threat that globally pervasive Cyanobacteria Harmful algal blooms (CyanoHABs) pose to humans. Major attention has been focused on the cyanobacterial hepatotoxin microcystins (MCs); however, there is a dearth of studies on cyanobacterial neurotoxin anatoxins. In this study, we explored how an anatoxin-producing *Cuspidothrix issatschenkoi* strain responded to culture with inorganic and organic nitrogen sources in terms of growth and anatoxins production. The results of our study revealed that ʟ- alanine could greatly boost cell growth, and was associated with the highest cell productivity, while urea significantly stimulated anatoxin production with the maximum anatoxin yield reaching 25.86 μg/mg dry weight, which was 1.56-fold higher than that in the control group (BG11). To further understand whether the carbon/nitrogen balance in *C. issatschenkoi* would affect anatoxin production, we explored growth and toxin production in response to different carbon/nitrogen ratios (C/N). Anatoxin production was mildly promoted when the C/N ratio was within low range, and significantly inhibited when the C/N ratio was within high range, showing approximately a three-fold difference. Furthermore, the transcriptional profile revealed that *anaC* gene expression was significantly up-regulated over 2–24 h when the C/N ratio was increased, and was significantly down-regulated after 96 h. Overall, our results further enriched the evidence that urea can stimulate cyanotoxin production, and ʟ-alanine could boost *C. issatschenkoi* proliferation, thus providing information for better management of aquatic systems. Moreover, by focusing on the intracellular C/N metabolic balance, this study explained the anatoxin production dynamics in *C. issatschenkoi* in response to different N sources.

## 1. Introduction

Cyanobacteria harmful algal blooms (CyanoHABs) have now become a universal issue threatening human and environmental health with increasing prevalence, intensity, and toxicity [1]. CyanoHABs’ frequent outbreak has affected the balance of the aquatic system, limited the availability of water resources in lakes and threatened the health of human and aquatic organisms and the sustainability of society. Among all these adverse impacts of CyanoHAB, cyanotoxins are of prime importance due to their detrimental effects on the human body. Cyanotoxins are generally categorized into four types: hepatotoxins, neurotoxins, dermatoxins, and cytotoxins [2]. Extensive studies have investigated the hepatotoxin microcystins (MCs) and their main producer *Microcystis* spp. [3,4,5]. The neurotoxin anatoxin-a has been detected at an increasing rate in freshwater bodies worldwide, including in Israel [6], Argentina [7], Berlin [8], the United States [9], and New Zealand [10]. There is no doubt that anatoxin-a in the environment poses a threat to livestock, wildlife, and would lead to human fatalities, but in different ways than MCs.

Anatoxin-a (ATX-a) and its analogue homo-anatoxin-a (HATX-a) (collectively referred to as anatoxins in this study) are low-molecular-weight neurotoxic alkaloids produced by planktonic and benthic cyanobacteria such as *Anabaena* [11], *Aphanizomenon* [12], *Oscillatoria* [13], *Phormidium* [14], and *Tychonema* [15]. Anatoxins are potent agonists of the nicotinic acetylcholine receptor, causing muscle cell overexcitation and symptoms of cramping, convulsion, and respiratory paralysis, and can provoke acute animal death [16,17]. A study reported that mice intraperitoneally injected with ATX-a and HATX-a had LD_50_ values ranging from 200–250 μg/kg [18]. Casualties caused by anatoxin intoxication were first reported in the early 1930s and have been constantly reported ever since [19,20,21,22].

Due to growing concerns about the detrimental neurotoxicity of anatoxins, numerous studies have been carried out to obtain a deeper understanding of anatoxins both at the molecular and physiological levels. Méjean et al. [23] deduced that ATX-a and HATX-a biosynthesis starts from ʟ-proline; they also proposed the steps involved in anatoxin-a biosynthesis [24,25,26,27]. Moreover, the anatoxin biosynthesis gene cluster has been identified in a few anatoxin-producing strains, including several *C. issatschenkoi* strains, *Oscillatoria* PCC 6506, and *Anabaena* sp. 37 [28,29,30,31]. Compared with MCs, knowledge of how abiotic factors might affect anatoxin production in anatoxin-producing strains is insufficient. Only in the past decade has there been an increase in research in this area, and recent studies have explored the role of physicochemical parameters, including light intensity, temperature, phosphorus concentration, nitrate concentration, and forms of nitrogen in anatoxin production by cyanobacteria [32,33,34,35,36].

It is believed that there is a strong correlation between nutrient over-enrichment and the persistence of CyanoHABs. One of the main elements that drives eutrophication is nitrogen, which occurs in excess due to anthropogenic loads of bioavailable N [37]. Nitrogen is not only essential in cell growth, but it plays a significant role in physiological processes such as toxin synthesis, including the synthesis of anatoxins. Both dissolved inorganic nitrogen (DIN) and dissolved organic nitrogen (DON) commonly exist in aquatic systems. Cyanobacteria have evolved the capacity to utilize both inorganic and organic N sources as nitrogenous nutrient sources, including ammonium, nitrate, nitrite, and urea [36,38,39,40]. Some cyanobacteria are able to fix N_2_ in the atmosphere [41,42]. In addition, cyanobacteria have been reported to have the ability to assimilate certain amino acids [40,43]. When there is thick bloom, DIN in water is exhausted quickly, leaving DON as the main nitrogen source for cyanobacteria [44]. In the past four decades, there has been a significant shift from nitrate- or ammonium-based fertilizers towards urea-based fertilizers in agriculture activities, and urea has now become a ubiquitous fertilizer accounting for more than 50% of global nitrogenous fertilizer usage [45]. The urea content in water bodies has increased along with this shift. Moreover, urea has been reported to stimulate algal growth and toxin concentration [46,47,48]. In addition to urea, amino acids naturally occur abundantly in water bodies, and have been reported to affect microcystin production in *Microcystis aeruginosa* [43]. However, most studies have only investigated the role of DIN; much less knowledge is available on the effect of DON, such as the effect of urea and free amino acids on the productivity and toxin synthesis of cyanobacteria, and considerably less is known about the role of DON in the production of cyanotoxins, other than MCs, in cyanobacteria. From this perspective, more efforts need to be made towards exploring whether DON can be a potential driver of cyanobacterial bloom and, particularly, anatoxin production.

*Cuspidothrix issatschenkoi*, formerly *Aphanizomenon issatschenkoi* [49], is an anatoxin producer that has been frequently isolated from water bodies where anatoxin-related intoxication incidents have been reported [12,50,51,52]. *C. issatschenkoi* occurs occasionally in the northern temperate zone but rarely forms blooms [29]. However, dense blooms of *C. issatschenkoi* were spotted in the southern temperate zone in New Zealand [52]. Though *C. issatschenkoi* blooms are relatively infrequent, it is well-known for its ability to produce anatoxin-a and its analogues. Anatoxin-producing *C. issatschenkoi* strains have been reported to occur in China, Germany, Japan, and New Zealand [12,28,52,53]. In Japan, *C. issatschenkoi* has been deemed to be the second commonest toxic strain after microcystin-producing *Microcystis aeruginosa* [54]. Thus, this species has received considerable attention due to its anatoxin-producing potential. However, no study has explored how *C. issatschenkoi* responds physiologically to different forms of N sources.

The aim of the present study was to investigate how different forms of DIN (nitrate and ammonium) and DON (urea, L-alanine, and L-proline) would affect the growth and anatoxin production in *C. issatschenkoi*. Additionally, this study attempted to explain the differences in anatoxin production induced by N sources from the perspective of the C–N balance in algal cells.

## 2. Results

### 2.1. C. issatschenkoi Cell Growth and Anatoxin Production When Cultured with Different N Sources

**Figure 1:**
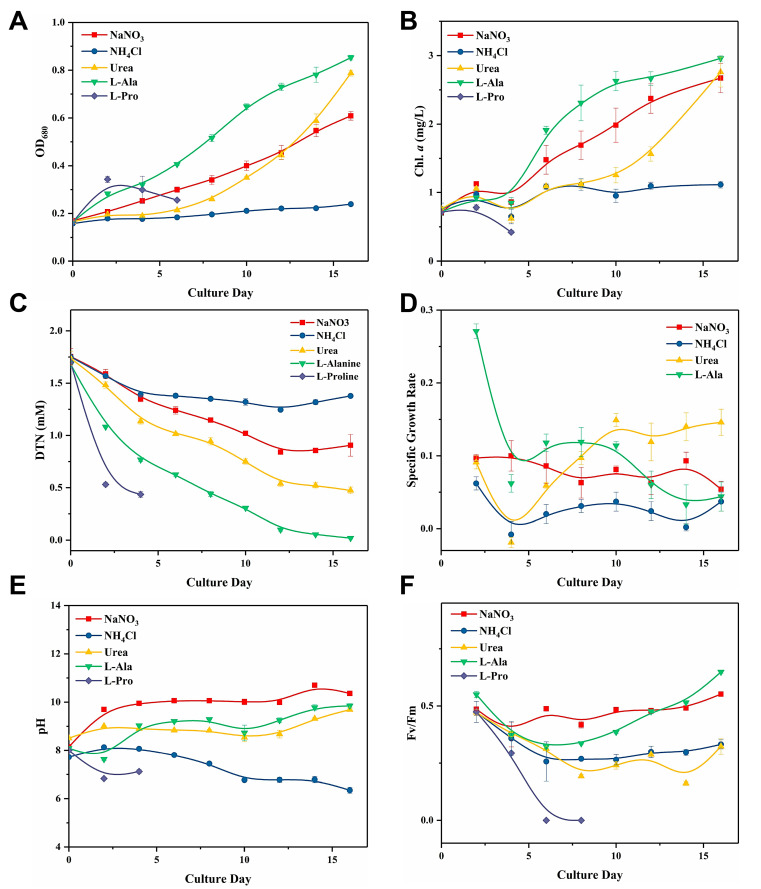
Effect of different forms of N sources on the (**A**) OD_680_, (**B**) Chlorophyll *a* (Chl *a*) concentration, (**C**) dissolved total nitrogen (DTN) consumption, (**D**) relative growth rate, (**E**) pH, and (**F**) F_v_/F_m_ of *Cuspidothrix issatschenkoi*.

Cells were cultivated with different N sources for 16 days and key parameters reflecting the cell growth states were measured. Figure 1A,B show the OD_680_ (optical density at 680nm) and chlorophyll *a* concentration, which exhibited a similar trend. Figure 1C shows the dissolved total nitrogen (DTN) consumption in the culture medium. The speed at which each N source was consumed ranked as follows: ʟ-alanine > urea > NaNO_3_ > NH_4_Cl (ʟ-proline was excluded). Obviously, cells could not grow normally in ʟ-proline or NH_4_Cl (1.76 mM-N). Obvious cell lysis was observed in the ʟ-proline group four days after inoculation. The biomass in the NH_4_Cl group showed nearly no increase, and there was a decline in DTN only on day 0–4. The NaNO_3_ group was considered as the control. The biomass in the ʟ-alanine group was maintained at the highest level almost throughout the whole culture duration, and it reached a final OD_680_ of 0.853 ± 0.01 on Day 16. ʟ-Alanine was consumed at the highest rate compared to all the other N sources (Figure 1C). However, although the ʟ-alanine group had the highest specific growth rate during the early period of cultivation, its growth rate drastically declined to a relatively low level after the maximum specific growth rate was reached on day 8 (Figure 1D). Cells in the urea group went through a six day lag phase, while no stagnation was observed in other groups. Then cells entered the logarithmic phase with a marked surge in specific growth rate and reached the second highest biomass (OD_680_ = 0.788 ± 0.012) on day 16.

The pH in normally growing groups was significantly higher than that in growth-inhibited groups (Figure 1E, Table 1), and the pH in the NH_4_Cl group decreased into the acidic range after day 10. The F_v_/F_m_ was found to manifest the maximum quantum yield for primary photochemistry (Figure 1F). Except for the NaNO_3_ group, the F_v_/F_m_ showed a decline in all the other groups during the preliminary culture period. However, the F_v_/F_m_ in the l-alanine groups returned to normal level as the experiment proceeded. Photosynthetic activities in the NH_4_Cl and l-proline groups were inhibited to different degrees. F_v_/F_m_ could not be detected in the l-proline group from day 6 onwards, whereas F_v_/F_m_ in the NH_4_Cl group remained consistently low throughout cultivation.

**Figure 2:**
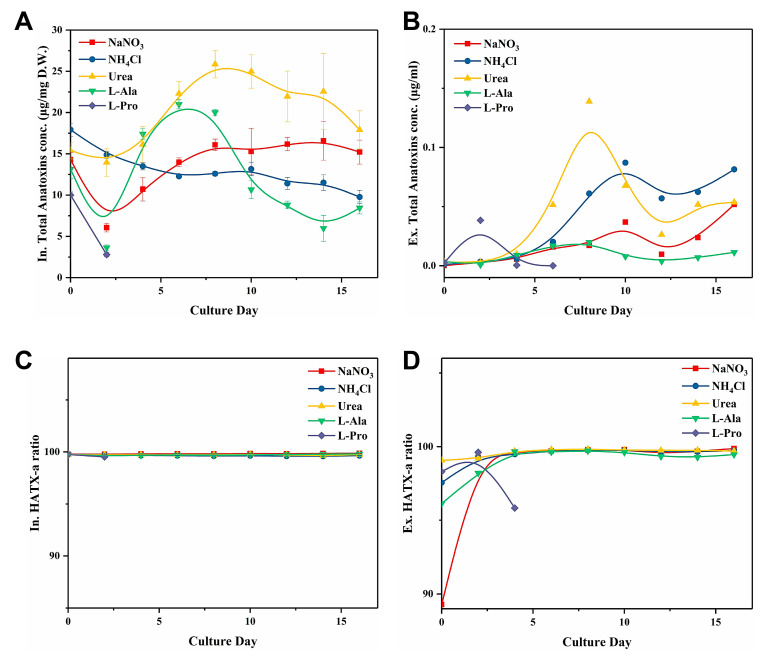
Effect of different types of N sources on the (**A**) Total intracellular anatoxin concentration, (**B**) Total extracellular anatoxin concentration, (**C**) Intracellular HATX-a ratio, and (**D**) Extracellular HATX-a ratio of *C. issatschenkoi*.

Regarding the toxin production dynamic of *C. issatschenkoi*, the intracellular gravimetric anatoxin concentration (Figure 2A) varied with the cell growth cycle. Cells in the urea group produced a maximum of 25.86 μg/mg dry weight (DW) on day 8, which was significantly higher than the corresponding toxin yield in other groups (*p* < 0.05; Table 1). The toxin concentration in the l-alanine group peaked on Day 6 (20.99 μg/mg DW) and then drastically dropped to the lowest level among all the groups. The increase in the total anatoxin concentration in the NaNO_3_ group slowed down after day 8 and fluctuated between 15–16 μg/mg DW. Toxin production in the NH_4_Cl group decreased consistently throughout the culture period, and the same trend was observed for the L-proline group.

The total volumetric concentration of extracellular anatoxins in all groups did not exceed 0.2 μg/mL (Figure 2B). However, an upward trend was observed in all groups during the late cultivating period. Overall, extracellular total anatoxin concentration in the late cultivating period could be ranked in decreasing order as follows: NH_4_Cl > urea > NaNO_3_ > L-alanine > L-proline.

The respective proportion of two anatoxin-a analogues (Figure 2C,D) remained stable across different N sources treatment, except for the HATX-a proportion in the extracellular toxin samples during the first two days of culture. Both intracellular and extracellular HATX-a proportions were stable, and HATX-a constantly accounted for over 99% of the total toxin yield. Therefore, a variation in the N source would not cause a shift in the HATX-a proportion.

### 2.2. C. issatschenkoi Cell Growth and Anatoxin Production under Different Urea Concentrations

**Figure 3:**
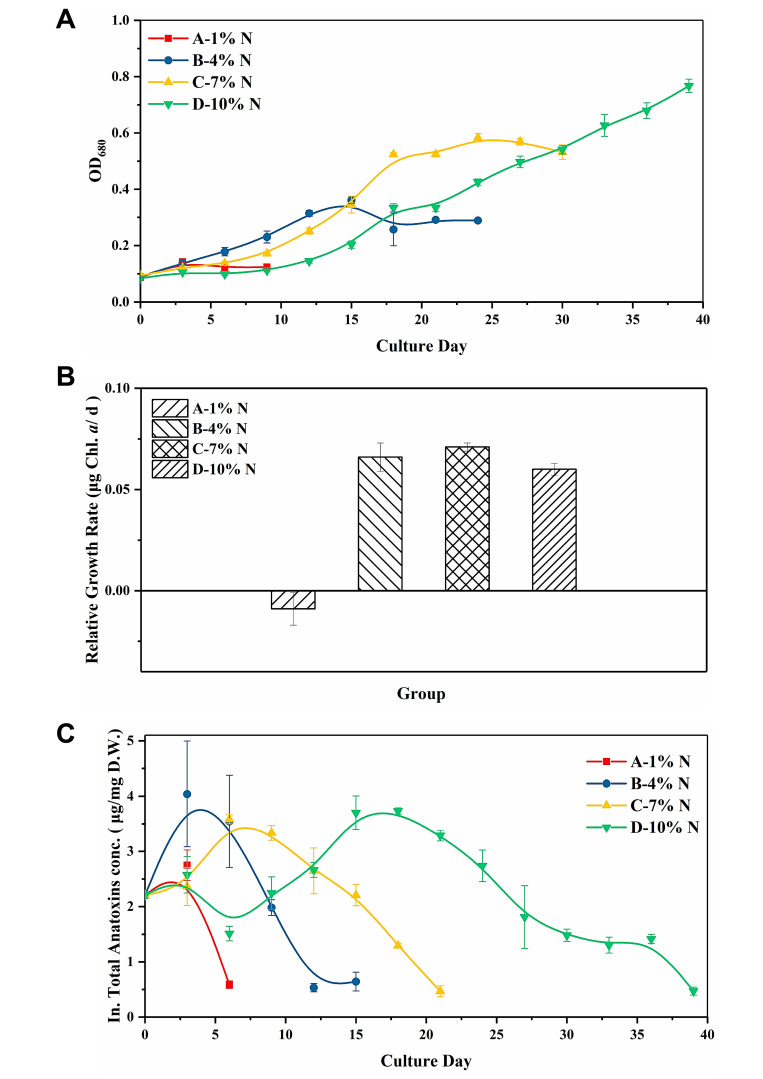
Effect of different urea concentrations on the (**A**) OD_680_, (**B**) relative growth rate, and (**C**) total intracellular anatoxin concentration of *C. issatschenkoi*. The N concentration in groups A–D corresponded to 1%, 4%, 7%, and 10% of the N concentration in BG11, which was 0.176, 0.704, 1.232 and 1.76 mM, respectively.

Figure 3A presents the result of the cell growth under different urea concentrations. Except for group A, which was treated with the lowest urea concentration (0.176 mM), normal growth could be seen in all the other groups. An increase in the urea concentration resulted in obvious extension of the lag phase, and it would take longer for cells to enter the plateau phase. Moreover, a higher urea concentration would lead to higher biomass yield, but there was no significant difference in the relative growth rate (Tukey’s honestly significant differences [HSD] test, *n* = 3, *p* > 0.05; Figure 3B).

Figure 3C shows the total intracellular anatoxin production dynamic. Anatoxin yield variations showed a similar trend in groups B–D, and only the time at which anatoxin production peaked varied. The respective maximum anatoxin yield achieved in groups B, C, and D was 4.038, 3.581, and 3.730 μg/mg DW, showing no significant difference (Tukey HSD test, *n* = 3, *p* > 0.05).

### 2.3. Cell Growth and Anatoxin Production of C. issatschenkoi under Different Initial Extracellular C/N Ratios

**Figure 4:**
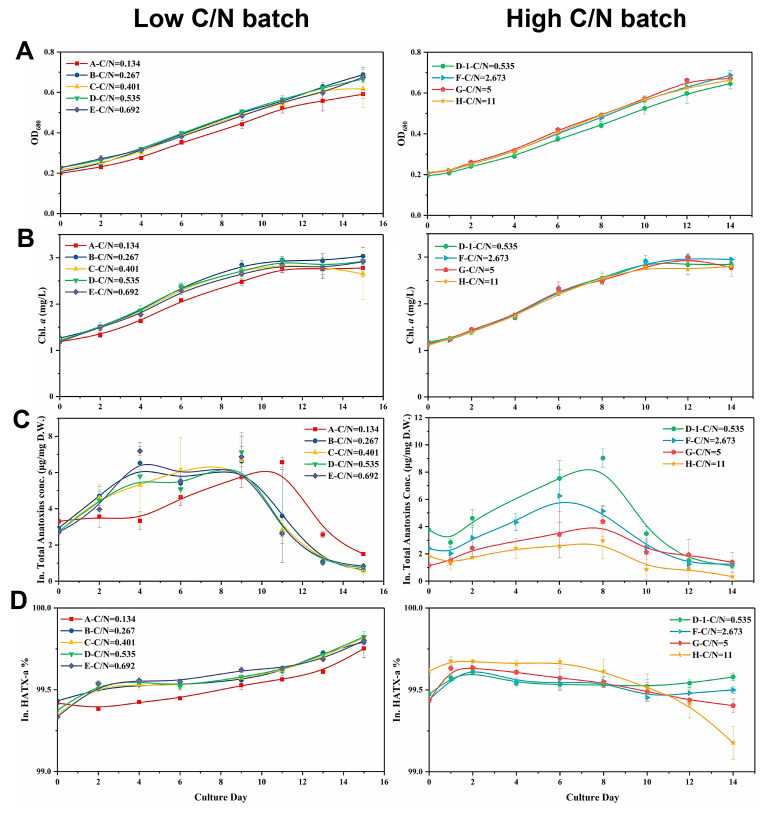
Changes in (**A**) OD_680_, (**B**) Chlorophyll (Chl) *a* concentration, (**C**) Total intracellular anatoxins concentration, and (**D**) HATX-a proportion when cells were cultured under different exogenous C/N ratios. The left row represented the batch with low C/N ratios, and the right row represented the batch with high C/N ratios.

Figure 4A,B show the growth curves in the two C/N ratio batches. In the low C/N batch, the OD_680_ and Chl *a* concentration was the lowest in group A. Correspondingly, the DIN consumption in group A was slower than that in the other groups within this batch (Figure S1), where the extracellular DIN of group A was almost exhausted on day 11, which was two days later than the other four groups. The extracellular DIN consumption rate in groups B–E was nearly identical. Therefore, low inorganic C supply could decelerate the uptake of DIN. In the high C/N batch, cell growth was not significantly affected by the increase in the C/N ratios (*p* > 0.05).

The effect of the C/N ratio on anatoxin production in *C. issatschenkoi* was range-dependent. In the low C/N batch (Figure 4C), anatoxin production in group A experienced stagnation for four days, resulting in lower toxin yield in this group relative to that in groups B–E during the early stage of cultivation, the difference of which was especially prominent on day 4 (*p* < 0.05). Toxin synthesis in this group resumed after the stagnation and peaked at 6.57 μg/mg DW on Day 11, which was two days later than the peak in the other groups. No stagnation in anatoxin synthesis was observed in groups B–E, showing a similar trend in variation. Moreover, the total intracellular anatoxin concentration rapidly declined in all the groups after the maximum toxin yield was reached. Interestingly, the timing of quick decomposition of anatoxins in each group coincided with the time when the extracellular DIN was almost completely exhausted (Figure S1).

In the high C/N batch, anatoxin synthesis was significantly down-regulated with the increase in C/N ratios (Figure 4C). Although there was a similar pattern in the variation trend across all the groups, their rangeabilities were largely affected by C/N ratios. At most of the sampling points, the groups in the high C/N batch could be ranked in descending order of anatoxin concentrations as follows: group D–1 > F > G > H. Obvious anatoxin decomposition could also be observed in the late growth phase, which was similar to that seen in the low C/N batch.

The HATX-a concentration in all the toxin samples from both batches accounted for over 99% of the total intracellular anatoxin yield (Figure 4D).

**Figure 5:**
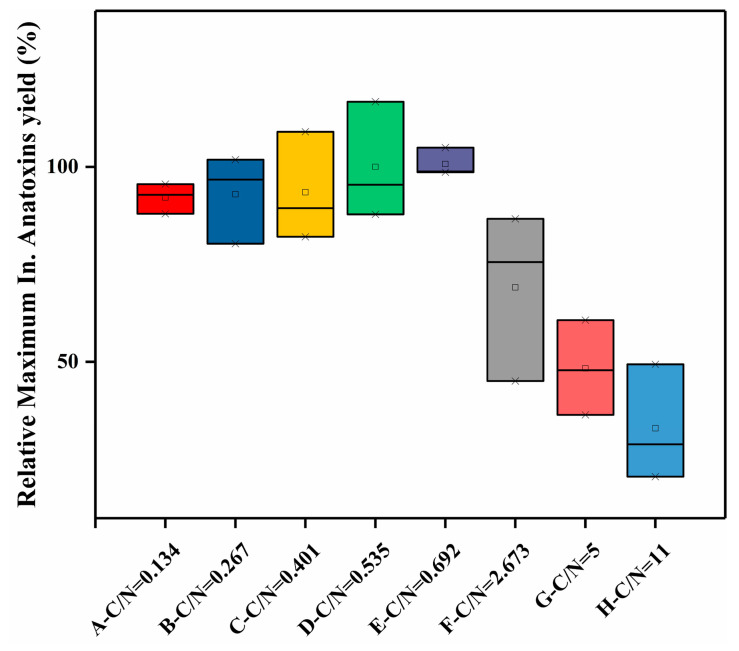
Maximum anatoxin yields in the C/N experiment. Squares represent the mean values; the upper crosses represent the maximum values; the lower crosses represent the minimum values; the black lines inside the boxes represent the median values.

In terms of the maximum intracellular anatoxin yield (Figure 5) in both the high and low C/N batches, a mild increasing trend could be observed in the low C/N batch, with the increase in C/N ratio, ranging from 6.57–7.07 μg/mg DW in groups A–E. On the other hand, the high C/N batch showed an opposite trend, with a significant decline in the maximum intracellular anatoxin yield with an increase in C/N ratios. In the high C/N batch, a nearly three-fold difference occurred between group D-1 (9.03 μg/mg DW) and group H (2.975 μg/mg DW). Thus, elevation in the C/N ratios within low C/N range would promote anatoxin production, whereas elevation within a high C/N range would lead to inhibition in anatoxin synthesis.

### 2.4. Relative anaC Expression Level under Different Initial Extracellular C/N Ratios

**Figure 6:**
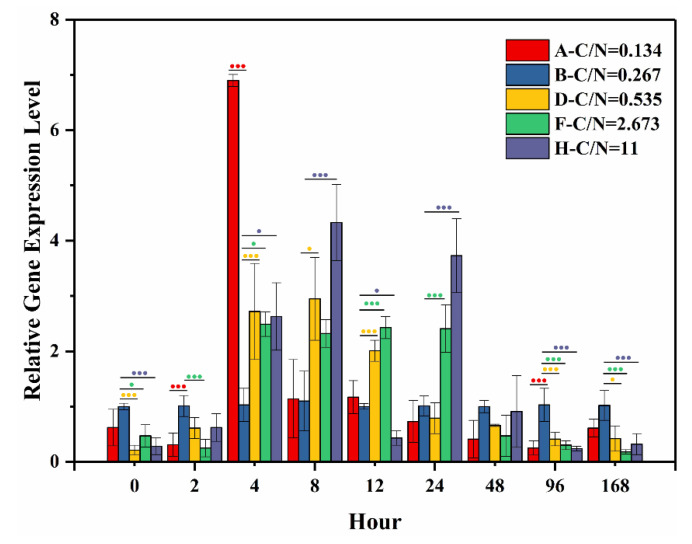
Relative expression level of *anaC* treated with different initial ratios of exogenous C/N. Significant difference between the control (group B) and each treatment group was tested using the Dunnett test (one-way ANOVA, *p* < 0.05), and was displayed by dots. One dot indicates significant difference (*p* < 0.05), and three dots indicate highly significant difference (*p* < 0.01). Colors of the dots match with each treatment group tested.

Figure 6 presents the relative gene expression level of *anaC*. Statistical analysis was conducted with group B set as the control (one-way ANOVA, Dunnett test, *p* < 0.05). For the first two hours of culture, significant down-regulation was observed. However, from 2–24 h, the *anaC* expression was significantly up-regulated in groups treated with higher C/N ratios compared with the control in almost every sampling hour, with groups D–H up-regulated by 2.01–2.69, 2.38–2.43, and 2.55–3.95 times the control, respectively. At 48 h, there was no significant difference between the control and treatment groups. After 96 h, the *anaC* expression was significantly down-regulated in the groups treated with higher C/N ratios compared to control. Specifically, the *anaC* expression in groups D–H was down-regulated 2.51, 3.39, and 4.36 times the control at 96 h and 2.46, 5.69, and 3.23 times the control, respectively, at 168 h.

## 3. Discussion

This is the first study to investigate how *C. issatschenkoi* growth is affected by culture in the presence of different N sources. ATX-a biosynthesis starts from proline [23]; however, *C. issatschenkoi* failed to survive when cultured with l-proline (1.76 mM-N) being the sole N source, let alone the possibility of its fueling ATX-a biosynthesis in cells. Hare et al. [55] found that *Arabidopsis* plants exposed to exogeneous proline (5–20 mM) suffered from visible chlorosis and ultrastructural damage; a similar phenomenon was encountered in our study. Since few studies have investigated how exogeneous proline affects the growth of cyanobacteria, it is speculated that exogenous L-proline (1.76 mM-N) might be toxic to *C. issatschenkoi*.

Another amino acid explored in our study was L-alanine. L-Alanine is an amino acid that has been reported to exist in natural waters with high abundance [56]. L-Alanine has a low molecular weight and it has been reported that it can be assimilated by cyanobacterial cells at a high rate [57]; this is consistent with our study, finding that *C. issatschenkoi* consumed l-alanine with relatively high speed. In turn, l-alanine greatly boosted cell growth and might add to the potential risk of cyanobacterial bloom outbreak, to which more attention should be drawn.

In most scenarios, ammonium is expected to trump other N sources for cyanobacterial growth as it can be used directly and in an energy-efficient manner [58]. However, the presence of ammonium at high concentrations as the sole or dominant N source might lead to inhibition of cyanobacterial growth [59]. The degree of inhibition of algal cells by ammonium varied [60]. Cell lysis would occur immediately or within hours if the ammonium concentration exceeded the level of tolerance by algae, or it would retard the cell growth rate, which was observed in our study. One of the indirect mechanisms by which ammonium exerts its toxicity is the variation in pH brought about by ammonium addition, and a low pH is considered an unfavorable condition for algal growth [61], which was consistent with the fact that the pH in the NH_4_Cl group gradually decreased to the acidic range.

Urea has been widely reported to stimulate cell productivity, photosynthetic activities, and toxin production in cyanobacteria [48]. However, inhibition in cell growth and photosynthetic activities of *C. issatschenkoi* were observed during the preliminary culture period. The stagnation could be explained via two aspects. First, it is energy-consuming for cells to hydrolyze urea with the urease complex. Second, studies have demonstrated that a high urea concentration would inhibit the growth of cyanobacteria [46,62]; in particular, elevation of urea concentration has been shown to be associated with an elongated lag phase. Despite these obstacles, *C. issatschenkoi* alleviated the negative effect accompanying the high urea concentration, showing great proliferation potential, and is possibly a relevant detoxification mechanism to export the excessive intracellular urea [63].

The toxic effects of anatoxins on mammals and macrophytes have been actively studied [64,65]; however, the biological role of anatoxin-a within algal cells has not been exclusively elucidated. Extensive studies supported that microcystins function in various ways to help *Microcystis* spp. gain competitive strength, especially when cells were exposed to adverse conditions [66,67,68]. In our study, anatoxin production did not increase when cell growth was obviously inhibited (due to the addition of ammonium or l-proline), suggesting that anatoxins cannot function as a stress-resistant substance when conditions for cell growth are not favorable.

Based on the anatoxins profile in this study, intracellular anatoxins were only accumulated under the premise of favorable cell growth. The anatoxin yield in the NH_4_Cl group, which suffered from ammonium toxicity, showed a constant decline throughout the culture period. Cells might assign priority to neutralizing the detrimental effect of high concentrations of ammonium by excluding it, which is a process that features high energy consumption, but is futile [69,70]. With energy spent on relevant processes, cells were already deficient in the energy needed to sustain normal anatoxin production. On the other hand, exogenous N in the NH_4_Cl group showed nearly no decline. Consequently, the constant decline in anatoxins might be the outcome of cells’ decomposing intracellular anatoxins, in order to compensate for the energy shortage. Nevertheless, the decline in anatoxin concentrations in the urea/l-alanine groups occurred only during the latter half of the culture period, when exogenous N was exhausted and cells were possibly faced with N limitation. Based on the observations above, it is speculated that anatoxins might function as an N-storage substance in algal cells. Studies have demonstrated that N-rich photosynthetic pigments in algal cells were catabolized when cells suffered from N deficiency [71,72]. However, no decline in Chl *a* was observed until day 16 when the exogenous N was almost exhausted, while anatoxins in the l-alanine group already showed dramatic decrease after day 8 when the exogeneous N supply in this group was relatively low. In addition, in the low C/N batch experiment, the time when the intracellular anatoxins concentration in each group showed a dramatic decline coincided with the time when their exogenous N supply was almost depleted, while there was no decrease in the biomass in each group. Therefore, not only might anatoxins function as a N reserve, but they may also be more sensitive to the availability of exogenous N than N-rich photosynthetic pigments.

Among the five given N sources, the anatoxin yield in the urea group exceeded those in the other groups at almost every sampling point. Urea has been reported to stimulate toxin production in cyanobacteria [73,74,75,76]. The urea transported to cyanobacterial cells would be decomposed by urease into ammonium and CO_2_, and the CO_2_ generated during this process was previously thought to be useless [77]. Yet Krausfeldt et al. [78] used isotope labeling and found that urea could function as both a nitrogen and carbon source in cyanobacterial growth, indicating that cells cultured with urea might have the advantages of an extra carbon source supply compared with cells cultured with inorganic N sources. As the N supply in each group was set to be the same, it is plausible to infer that the advantages that the urea group exhibited in toxin yield might be due to the contribution of extra C derived from urea hydrolysis. In respect to the relation between cell growth and anatoxin production in the urea group, it was noticed that the biomass in the urea group did not show obvious predominance until the end of the culture period. However, its anatoxin yield exceeded that of other groups even after day 2, when the cell growth was obviously stagnant. A similar outcome was also observed when cells were treated with different concentrations of urea. Finlay et al. [76] found that urea addition did not alter *Microcystis* abundance, but it could significantly stimulate microcystin production. Taken together, all this might support the speculation that the C and N generated from urea hydrolysis would directly fuel key physiological processes other than cell growth, such as pigment synthesis and toxin production [79]. Further investigation should focus on how the C and N from urea flux in cell metabolism, especially the allocation in cell growth and toxin synthesis, to interpret the mechanism underlying this phenomenon.

In the N source experiment, groups could be ranked according to their maximum intracellular gravimetric anatoxins quota as follows: urea > L-alanine > NaNO_3_ > NH_4_Cl > L-proline. To sum up, with the l-proline group excluded, the anatoxins yield was higher in the organic N groups than in the inorganic N groups. Chemically, DON differs from DIN in that DON is composed of extra carbon atoms. Hence, uptake of DON would lead to a shift in the carbon/nitrogen balance state in cells. To further explain the differences in the observed anatoxin yield in the N source experiment, the C/N experiment was conducted to simulate the C/N range in the N source experiment.

With respect to the specific anatoxin yield, our result suggested that the C/N ratio might have a range-dependent dual role in anatoxin production. Anatoxin production in *C. issatschenkoi* was found to be promoted with the increase in C/N ratio within low C/N range, although this change was not significant. However, an elevation in the C/N ratio would lead to significant up-regulation of *anaC* expression during the first 24 h, indicating that the induction of a higher C/N ratio would indeed stimulate anatoxin synthesis at the transcriptional level. On the other hand, anatoxin synthesis would be obviously suppressed when the C/N ratio is within relatively high range. From 96 h of culture, *anaC* expression in groups with higher C/N values than the control was found to be significantly down-regulated, which could explain the significant suppression in anatoxin production observed in the high C/N batch experiment.

Taken together, these results were consistent with the outcomes of anatoxin yield obtained from the N source experiment. Specifically, the C/N ratio in the organic N groups is higher than that in the inorganic N groups, and anatoxin yield in the inorganic N group was lower than that in the organic N group. Within the organic N group, on the other hand, the anatoxin yield in the urea group was higher than that in the l-alanine group, while the C/N ratio in the urea group was lower than that in the L-alanine group.

It was inferred that there might exist a C/N balance point for anatoxin production, and either high or low C/N ratios would disrupt this balance. Cyanobacteria have specific transport systems for active N uptake apart from passive diffusion. Nitrogen sources would first be converted to ammonium through specific metabolic processes before being exploited by algal cells. Ammonium assimilated in various ways would then be incorporated into carbon skeletons mainly through the sequential operation of two enzymes, glutamine synthetase (GS) and glutamate synthase (GOGAT), commonly known as the GS–GOGAT pathway [80]. The carbon skeleton required for ammonium incorporation is provided by 2-oxoglutarate (2-OG), which has been reported to function as a signaling metabolite of the C/N state [81,82]. Levitan et al. [83] reported that enriched CO_2_ enhanced the C/N ratio, N fixation, and biomass of *Trichodesmium*. Acceleration in DIN consumption caused by elevation in the C/N ratio was also observed in our study. However, when the C/N ratio was too high and disrupted the balance of C/N metabolism, which was the case of the high C/N batch in this study, cells would sense the imbalance and were deemed to be N-limited or C-oversupplied. Subsequently, the 2-OG in cells would increase in order to function in different ways: to simultaneously up-regulate nitrogen assimilation and down-regulate carbon uptake [84] in order to regain the balance of carbon/nitrogen metabolism. For example, biosynthesis of a C-rich compound such as carotenoid and astaxanthin would be up-regulated when the C/N ratio is high [85,86]. Meanwhile, studies have supported that the biosynthesis of N compounds would be reduced, and increasing recycling of N compounds has also been observed [87]. Although anatoxins only have one N atom and should be considered C-rich compounds, N deprivation directly limits their synthesis (Buckets Effect). In our study, a decline in the anatoxin quota might also be the result of the recycling of N compounds when the C/N ratio was high, which is consistent with the speculation that anatoxins might function as a sensitive N reserve in algal cells. It was also inferred that there might exist certain signaling substances coupling the N deprivation signal with the catabolism of anatoxins.

Utilization of inorganic and organic N would surely alter the C/N ratio in cells, and the uptake of different N sources would not simply lead to the same effect on the cell’s C/N metabolism, as the assimilation of various N sources would differ in form and efficiency. The C/N experiment aimed to simulate the variation in C/N values caused by different N sources. Although it was not identical to the exact mechanism by which different N sources would affect anatoxin production, it nevertheless provided us with more information from a general perspective.

Extracellular anatoxins concentrations were consistently low across the different N treatment groups, and generally did not exceed 0.2 µg/mL. Extracellular anatoxin concentration in all groups during late the cultivating period showed an upward trend, a phenomenon frequently observed when cell growth was approaching plateau phase due to poor algal growth state and increasing release of the toxin [88,89]. The low extracellular toxin concentration might be related to the unstable properties of anatoxins under the conditions of high pH, non-sterile conditions, photosynthetically active radiation (PAR), high temperature, and high UV-B irradiation [90,91]. Except for the NH_4_Cl and L-proline groups, the pH values in all groups increased to a high level, which is not favorable for anatoxin. Therefore, anatoxins released from cells might have already been rapidly degraded before measures were taken to stabilize them (by adding formic acid). This should remind researchers that better measures need to be taken to improve the accuracy of detecting anatoxin concentrations in natural water bodies. From a different perspective, the threats posed by anatoxins are mainly due to intracellular toxins, supported by the fact that potential anatoxin-producing algae cells and cell fragments were detected in the bodies of animals that died from anatoxin-poisoning incidents, even when anatoxin concentrations in the water column remained low. This could provide more insights into the prevention of anatoxin-related poisoning incidents.

## 4. Conclusions

Our study investigated how *C. issatschenkoi* responded to different forms of N sources (1.76 mM-N) in growth and anatoxin production. It was found that L-alanine would greatly boost the accumulation of *C. issatschenkoi*, which should draw more attention regarding the risk this may add to the outbreak of cyanobacteria bloom. Organic N sources would stimulate the anatoxin production in *C. issatschenkoi* compared with inorganic N sources, in which the most significant increase was observed in the urea group. Furthermore, C/N experiment helped us look into the possible reason why organic N source would enhance anatoxin production. Results showed that a higher C/N value would indeed promote anatoxin yield as well as the expression of *anaC* gene. However, when C/N value was too high to break the C/N metabolic balance in cells, anatoxin production would be significantly inhibited. This study provided more information for the management of aquatic systems, regarding the potential risks that anatoxin-producing *C. issatschenkoi* might have in response to different forms of N sources. In addition, based on monitoring of the detailed dynamics of intracellular anatoxin concentration, it was speculated that the intracellular anatoxins might act as a N-reserving substance sensitive to the ambient N status. Further investigations are needed to verify this speculation and find out the possible signaling mechanism and to help us gain better understanding about the biological role of anatoxins in algal cells.

## 5. Materials and Methods

### 5.1. Strain and Culture Conditions

All the experiments were performed with an anatoxin-producing *C. issatschenkoi* strain (FACHB-2454) provided by the Freshwater Algae Culture Collection at the Institute of Hydrobiology, Chinese Academy of Sciences (FACHB-Collection; Wuhan, China). This strain has lost its N_2_-fixing ability due to longtime preservation. Batch cultures were incubated with a light intensity of 30 µmol∙m^−2^∙s^−1^ on a 12:12 h diel cycle at 25 °C in 500 mL Erlenmeyer flasks. All the experiments were performed in triplicate, and the flasks were shaken 2–3 times and randomly replaced on a daily basis to avoid causing any effect on algal growth due to minor differences in the light intensity.

### 5.2. Experimental Design

Prior to each experiment, cells were first cultured with N-free BG11 medium [92] for 3 days to exhaust the N from algal cells without damaging them. Cells were then collected onto a 0.47-μm cellulose acetate membrane by filtration, and the cells were resuspended into corresponding growth media for subsequent experiments.

To explore the effect of different N sources on *C. issatschenkoi*, cells were inoculated into five different types of modified BG11 culture mediums with sodium nitrate (NaNO_3_), ammonium chloride (NH_4_Cl), urea (CH_4_N_2_O), L-alanine (C_3_H_7_NO_2_), and L-proline (C_5_H_9_NO_2_), as the sole N sources, respectively. The initial N concentration in the growth medium was set at 1.76 mM, which corresponded to 10% of the nitrogen concentration in the original BG11 medium. The initial OD_680_ was set at 0.2 in each treatment.

To further explore the effect of different urea concentrations on the anatoxin production in *C. issatschenkoi*, the cells were inoculated into BG11 medium containing urea instead of the original N source, with the final N concentration corresponding to 1%, 4%, 7%, and 10% of the N concentration in original BG11. The initial OD_680_ in each treatment was set at 0.1.

The C/N (molar ratio) experiment was performed to gain a better understanding of the differences in anatoxin production in the presence of various N sources. The C/N values in growth media were altered by adjusting the Na_2_CO_3_ concentrations in BG11 medium and the NaNO_3_ concentration was set at 0.706 mM, which corresponded to 4% of the original N concentration in the BG11 medium. The experiments were performed in two batches with a low C/N batch (0.134–0.692) and a high C/N batch (0.535–11). Table 2 displays the specific setting for growth media.

For all the experiments, the proper volume of samples was withdrawn every two or three days. Samples were taken to measure the optical density at 680 nm (OD_680_), pH, F_v_/F_m_, and extracellular N consumption, to extract and determine the chlorophyll *a*, ATX-a, and HATX-a concentrations.

### 5.3. Cell Growth, pH, N Consumption, and Photosynthetic Activity

The OD_680_ was measured using 1 mL of the samples from each flask in a UV spectrophotometer. The chlorophyll *a* (Chl *a*) concentration was determined via the acetone extraction method [93]. Briefly, 3–5 mL of the algal sample was filtered through a glass microfiber GF/C membrane (Whatman, GE Healthcare, Chicago, IL, USA), and the membrane was then soaked in 5 mL of acetone (80% *v*/*v*) and incubated at 4 °C in the dark. After 24 h, the tubes were centrifuged at 7000 rpm for 10 min at 4 °C, and the absorbances of the supernatants at 645, 663, and 750 nm were measured. The pH was measured directly with the samples using a pH meter.

The relative growth rate was calculated to assess the maximum biomass yield according to the following equation: μ_max_ = (C_1_ – C_0_)/(t_1_ – t_0_), where C_1_ and C_0_ represented the biomass when cells entered the plateau phase and the initial biomass, and t_1_ – t_0_ represented the number of days it took for the cells to enter the plateau phase.

The specific growth rate (μ) between two adjacent sampling points was calculated according to the following equation: μ = (lnC_t2_ – lnC_t1_)/(t_2_ – t_1_), where C_t2_ and C_t1_ are the biomass at the time of t_2_ and t_1_, respectively.

In the N source experiment, the DTN consumption was determined according to the UV Spectrophotometric Method-Alkaline Potassium Persulfate Digestion Method (GB 11894-89). NO_3_^−^ consumption in the C/N experiment was determined by measuring the OD_220_ in the algal culture filtrate. Sample filtrates were diluted to fit the standard curve if needed.

F_v_/F_m_ was measured using a Water-PAM fluorescence monitoring system (Walz, Effeltrich, Germany). First, the samples were placed in the dark for 10 min before measurement. Then, the minimum fluorescence (F_0_) was measured under a low light intensity and the maximum fluorescence (F_m_) was obtained under a saturating pulse. F_v_/F_m_ was calculated according to the following equation: F_v_/F_m_ = (F_m_ – F_o_)/F_m_.

### 5.4. Anatoxins Extraction

For analysis of the extracellular (Ex) toxin concentration, the algal sample was filtered through a 0.22-μm Millipore filter (Merck Millipore, Darmstadt, Germany). Then, 1 mL of the filtrate was collected, and 10 µL of formic acid was added to maintain the stability of anatoxins. The Ex toxin samples were stored at −20 °C before quantification.

To measure the intracellular (In) toxin concentration, algal cells were collected by filtration through 0.45-μm cellulose acetate membranes. The algae filaments were gently rinsed down into 2-mL pre-weighed Eppendorf tubes using milliQ water. The tubes were immediately frozen in liquid nitrogen and stored at −20 °C until use.

Before intracellular toxin extraction, algal cell samples were lyophilized, and the tubes were weighed again to calculate the dry weight of the collected cells. Then, 1 mL of methanol was added to each tube. The cells were lysed by three freeze-thaw cycles using liquid nitrogen and a 37 °C water bath, and the samples were shaken at 1200 rpm for 2 h using an Eppendorf thermomixer compact (Eppendorf, Germany). After centrifugation (12,000 rpm, 10 min), the supernatants were harvested to a new 2-mL Eppendorf tube. Another 1 mL of methanol was added to the algal cells to extract for another 1 h. The supernatants collected following two cycles of extraction were mixed and the methanol was volatilized by a vacuum centrifugal concentrator. Finally, 1 mL of 10% methanol with 0.1% (*v*/*v*) formic acid was added after centrifugation (14,000 rpm at 4 °C for 15 min) to re-dissolve the toxin. The supernatants were used to determine the toxin concentration.

### 5.5. Anatoxins Quantification

Since the *C. issatschenkoi* strain produces mostly (99%) HATX-a and trace (<1%) amounts of ATX-a, we used high performance liquid chromatography (HPLC) and ultra performance liquid chromatography (UPLC) separately to determine the concentration of these two analogues in each intracellular toxin sample to obtain data with higher precision. For extracellular toxin samples, we directly used UPLC-MS/MS to quantify the two analogues.

The high concentration of HATX-a was analyzed with an Alliance Waters™ e2695 (Waters, Milford, MA, USA) separation module coupled with a photodiode array (PDA) detector using a C18 cartridge (Prevail™ C18 Column, 250 × 4.6 mm, 5 µm, W. R. Grace and Company, Columbia, MD, USA). The column was maintained at 30 °C throughout the run. The mobile phase was acetonitrile (A) and 0.05% trifluoroacetic acid (TFA) (B) with the optimized gradient elution described as follows: linear gradient elution started with 10% of eluent A, held for 9 min, then increased to 90% eluent A over 1 min, held for 5 min, followed by a decrease to 10% eluent A over 1 min, and then held for 7 min until the next run. The total run time was 23 min. The flow rate was 1 mL/min. The injection volume was 10 μL. The HATX-a content was monitored at 227 nm by recording UV spectra from 200–400, and data were acquired via Empower 3.0 (Waters, Milford, MA, USA).

Low concentrations of ATX-a and HATX-a were analyzed using an Ultra-Performance Liquid Chromatography couple with a Tandem Quadrupole (Triple Quadrupole) Mass Spectrometry (ACQUITY UPLC H-class-Xevo TQ MS). An Acquity UPLC BEH C18 column (2.1 × 50 mm^2^, 1.7 μm) was operated at 30 °C. The mobile phase was methanol (A) and water (B), both with 0.1% formic acid. The elution conditions were as follows: linear gradient elution started with 5% of eluent A, followed by a linear gradient to 10% of eluent A in 3 min, increased to 80% eluent A over 0.2 min, held for 2.3 min, decreased to 5% over 0.2 min, and held for 1.3 min until the next run. The total run time was 7 min. The flow rate was 0.2 mL/min. The injection volume was 5 μL. The ATX-a standard was purchased from National Research Council, Canada, and the HATX-a standard was purchased from Novakits, France.

Gravimetric anatoxin concentration in intracellular toxin samples were calculated via dividing the total toxin content (μg) in the sample by the dry weight (mg) of the algae used for toxin extraction. Anatoxin concentration in the extracellular toxin samples was represented by volumetric concentration (μg/mL).

### 5.6. RNA Extraction, cDNA Synthesis, and Relative RT-qPCR Analysis of Gene Expression

Algal cells in groups A, B, D, F, and H from the C/N experiment were selected to quantify the relative expression level of *anaC* (the gene involved in the first step of anatoxin biosynthesis). Briefly, algal culture was harvested onto a 1.0-μm Nuclepore Track-Etch membrane (Whatman, Maidstone, Kent, UK) at 0, 2, 4, 8, 12, 24, 48, 96, and 168 h after inoculation. The filters were frozen in liquid N_2_ immediately and stored at −80 °C before RNA extraction. Total RNA was extracted using Trizol reagent (Invitrogen, Carlsbad, CA, USA). RNA purity and concentration were determined via a Nanodrop 8000 spectrophotometer (Thermo, Waltham, MA, USA). gDNA in the total RNA sample was removed, and 1 μg of total RNA was reverse transcribed into cDNA using a HiScript^®^ Q RT Supermix for qPCR kit (Vazyme Biotech, Nanjing, China). RT-qPCR was performed using the ChamQ SYBR qPCR Master Mix kit (Vazyme Biotech, Nanjing, China). Two pairs of primers were used to quantify the relative expression level of *anaC*. The forward and reverse primer sequences for *16S* rRNA were 5′-TAAGCATCGGCTAACTCC-3′ and 5′-ATTTCACCGCTACACCAG-3′ (201 bp). The forward and reverse primer sequence for *anaC* were 5′-AAGACCGCGTTTCCAGTCAT-3′ and 5′-CCGATAAAGCGGCTGAGAGT-3′ (111 bp). Data presented were the relative mRNA levels normalized against *16S* rRNA transcript levels, and group B was set as the control. RT-qPCR assays were performed in three biological replicates.

### 5.7. Statistical Analysis

Each experiment was conducted in triplicate. Data were presented as the means ± standard deviations. One-way analysis of variance (ANOVA) was used to assess statistical significance. In the statistical analysis involved in the N sources experiment and the C/N experiment, Tukey’s test was used and significance was determined at a *p* level of 0.05. Dunnett test was used to assess the significant difference in the relative expression level of *anaC* between the control group and each treatment group (*p* = 0.05).

## Figures and Tables

**Table 1:** Effect of different forms of N sources on the growth and anatoxin production of *C. issatschenkoi after* 16 days’ culture. Initial carbon/nitrogen (C/N) ratio in each treatment group was calculated and listed in the table.

Treatments	C/N Value	C (mM)	N (mM)	pH	F_v_/F_m_	Cell Productivity (mg Chl *a* L^−1^·d^−1^)	Maximum Intracellular Anatoxins Yield (µg/mg Dry Weight [DW])
NaNO_3_	0.107	0.189	1.765	9.886 ± 0.023 ^c^	0.471 ± 0.005 ^c^	0.123 ± 0.013 ^c^	16.572 ± 2.343 ^b^
NH_4_Cl	0.107	0.189	1.765	7.317 ± 0.038 ^a^	0.319 ± 0.004 ^b^	0.022 ± 0.003 ^b^	17.921 ± 0.787 ^b^
Urea (CH_4_N_2_O)	0.607	1.071	1.765	8.924 ± 0.054 ^b^	0.296 ± 0.008 ^b^	0.125 ± 0.014 ^c^	25.86 ± 1.667 ^c^
L-alanine (C_3_H_7_NO_2_)	3.107	5.483	1.765	8.985 ± 0.053 ^b^	0.451 ± 0.002 ^c^	0.140 ± 0.003 ^c^	20.993 ± 0.554 ^b^
L-proline (C_5_H_9_NO_2_)	5.107	9.013	1.765	7.318 ± 0.03 ^a^	0.143 ± 0.039 ^a^	−0.073 ± 0.006 ^a^	10.003 ± 0.368 ^a^

^a–c^ The values indicated by the same letter represented no significant difference, *p* = 0.05 by Tukey’s test.

**Table 2:** Effect of initial exogenous C/N ratio on cell proliferation and maximum anatoxin yield of *C. issatschenkoi*, and C and N concentrations in growth mediums with different C/N ratios.

Group		C/N Value	C (mM)	N (mM)	Cell Productivity (mg Chl *a* L^−1^·d^−1^)	Maximum Intracellular Anatoxins Yield (µg/mg DW)
Low C/N batch	A	0.134	0.094	0.706	0.145 ± 0.009	6.570 ± 0.272
	B	0.267	0.189	0.706	0.153 ± 0.007	6.631 ± 0.800
	C	0.401	0.283	0.706	0.147 ± 0.000	6.668 ± 0.989
	D	0.535	0.377	0.706	0.158 ± 0.012	7.131 ± 1.063
	E	0.692	0.489	0.706	0.151 ± 0.018	7.188 ± 1.147
High C/N batch	D-1	0.535	0.377	0.706	0.173 ± 0.012	9.030 ± 0.682 ^c^
	F	2.673	1.887	0.706	0.174 ± 0.006	6.243 ± 1.944 ^b^
	G	5	3.29	0.706	0.166 ± 0.002	4.364 ± 1.102 ^b^
	H	11	7.663	0.706	0.168 ± 0.002	2.975 ± 1.335 ^a^

^a–c^ Values indicated by the same letter represent no significant difference, *p* = 0.05 by Tukey’s test.

## Supplementary Materials

The following are available online at https://www.mdpi.com/2072-6651/12/11/724/s1, Figure S1: Dissolved inorganic nitrogen consumption in the low C/N batch.
