# Supplementary material for: Understanding the Differences in the Growth and Toxin Production of Anatoxin-Producing Cuspidothrix issatschenkoi Cultured with Inorganic and Organic N Sources from a New Perspective: Carbon/Nitrogen Metabolic Balance
Source: Toxins (Basel). 2020 Nov 19;12(11):724. doi: 10.3390/toxins12110724 (PMC7699347; doi:10.3390/toxins12110724)
Supplement: Supplementary file 1 [file toxins-12-00724-s001.pdf]

# Supplementary Materials: Understanding the Differences in the Growth and Toxin Production of Anatoxin-Producing *Cuspidothrix issatschenkoi* Cultured with Inorganic and Organic N Sources from a New Perspective: Carbon/Nitrogen Metabolic Balance

**Siyi Tao, Suqin Wang, Lirong Song and Nanqin Gan**

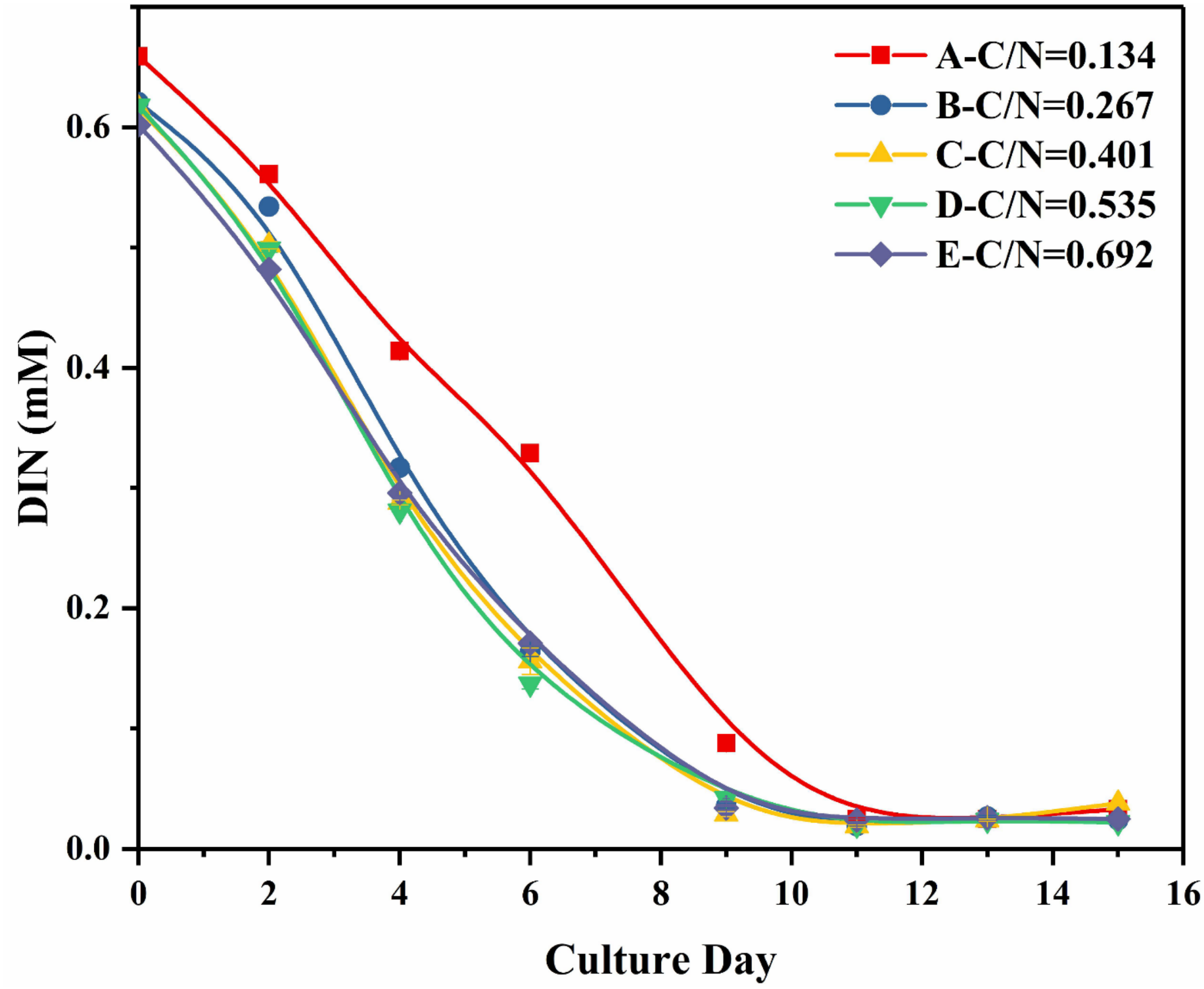


**Figure S1.** Dissolved inorganic nitrogen consumption in the low C/N batch.
